# Electrochemical, Electrochemiluminescence, and Photoelectrochemical Aptamer-Based Nanostructured Sensors for Biomarker Analysis

**DOI:** 10.3390/bios6030039

**Published:** 2016-08-02

**Authors:** Andrea Ravalli, Diego Voccia, Ilaria Palchetti, Giovanna Marrazza

**Affiliations:** Department of Chemistry “Ugo Schiff”, University of Florence, Via della Lastruccia 3, 50019 Sesto Fiorentino (FI), Italy; andrea.ravalli@unifi.it (A.R.); diego.voccia@unifi.it (D.V.); ilaria.palchetti@unifi.it (I.P.)

**Keywords:** aptamer, biosensor, electrochemical, electrochemiluminescent, photoelectrochemical, biomarker, nanomaterial

## Abstract

Aptamer-based sensors have been intensively investigated as potential analytical tools in clinical analysis providing the desired portability, fast response, sensitivity, and specificity, in addition to lower cost and simplicity versus conventional methods. The aim of this review, without pretending to be exhaustive, is to give the readers an overview of recent important achievements about electrochemical, electrochemiluminescence, and photoelectrochemical aptasensors for the protein biomarker determination, mainly cancer related biomarkers, by selected recent publications. Special emphasis is placed on nanostructured-based aptasensors, which show a substantial improvement of the analytical performances.

## 1. Introduction

Biomarkers are defined by the Food and Drug Administration (FDA) and the European Union National Institute of Health as a “characteristic that is objectively measured and evaluated as an indicator of normal biological processes, pathogenic processes, or pharmacologic responses to therapeutic intervention. Biomarkers act as indicators of a normal or a pathogenic biological process. They allow assessing the pharmacological response to a therapeutic intervention. A biomarker shows a specific physical trait or a measurable biologically change in the body that is linked to a disease or a particular health condition”. Briefly, if the concentration of a molecule (i.e., enzymes, protein, oligonucleotide acids, etc.) increases or decreases in biological fluids in relation to the presence or during therapeutic treatment of a disease, this molecule can be named a clinical biomarker [1]. Due to the high death rate and high psychological impact on the society of cancer disease, the attention of the researchers has been mainly focused on the discovery and the evaluation of cancer-related biomarkers.

Cancer biomarkers are classified according to the disease state, to the chemical nature of the molecule (DNA, RNA, protein) or to other parameters such as imaging, pathological, or in silico biomarkers [2]. Among all biomarkers, proteins represent the most studied molecules because they can be strictly related to the disease state [3].

The first cancer protein biomarker was the discovery in 1847 of the presence of a tumor-related free antibody light chain (named as Bence-Jones protein) in the urine of a patient affected with multiple myeloma cancer [4]. Successively, many cancer-associated proteins have been evaluated by in-vivo experiments by the immunization of laboratory animals. The specific cancer associated antigen is then recognized by the appropriated monoclonal antibodies in the extracts obtained from cancer tissues or cell lines. The complete sequencing of the human genome and the technological advances in the proteomic field led to recognition of new cancer biomarkers [5,6]. However, some limitations still need to be overcome, i.e., protein overexpression with more than one cancer process, false positive results, the ability to discriminate the different types and stages of cancer diseases. Nevertheless, a growing consensus suggests the construction of a cancer biomarkers panel in order to overcome these drawbacks [7]. In clinical assays, the conventional methods of detection of tumor markers in serum include immunological assays, time-resolved fluorescence, chemiluminescence, etc. These techniques have some disadvantages, such as being time-consuming, having poor precision, and experience difficulty in realizing automation. Therefore, there is an urgent requirement for the development of new assays with low-cost, high speed, and real-time control in large-scale disease screening [8].

In this context, biosensors have been extensively investigated as potential analytical tools providing multiplexed analysis, simplicity, fast response, sensitivity, and specificity, in addition to lower cost [9]. Aptamer-based sensors (aptasensors) appear promising among all biosensors because they combine all of these characteristics [10,11].

Aptamers are small, single-stranded DNA or RNA sequences isolated from a library of random sequences by an “in vitro” selection and amplification process, called SELEX (Systematic Evolution of Ligands by EXponential enrichment). They have a greater affinity for various analytes, like antibodies, acting as capture molecules. Their binding mechanism is mainly due to their three-dimensional stable structure that binds the target molecule. Their common conformational structures and chemo-physical features have been well discussed and reviewed in literature [12,13]. Due to their advantages respect to antibodies such as high stability, chemical synthesis, low-dimension aptamers have been successfully applied as bioreceptor in sensor technology. A wide variety of them has been applied in various fields [14,15,16].

Disease biomarkers are often present at ultra-low levels and require ultrasensitive methods for detection. Therefore, the coupling of aptamers and nanomaterials has been extensively exploited for sensing and biosensing. Nanomaterial applications can be classified into two groups according to their functions: (1) nanomaterial-modified transducers to facilitate bioreceptor immobilization or improve properties of transducers; and (2) nanomaterial-biomolecular conjugates as labels.

In this review, the recent reports regarding the development of aptamer-based biosensors for analysis of protein biomarkers, mainly cancer related biomarkers, using electrochemical, electrochemiluminescence and photoelectrochemical transduction are presented. Special emphasis is placed on nanostructured-based aptasensors, which show a significant improvement of the analytical performances. 

## 2. Discussion

### 2.1. Aptasensor Formats

A biosensor is defined by the International Union of Pure and Applied Chemistry (IUPAC) as “a self-contained integrated device which is capable of providing specific quantitative or semi-quantitative analytical information using a biological recognition element (biochemical receptor or bioreceptor) which is in direct spatial contact with a transducer. The transducer is used to convert (bio)chemical signal resulting from the interaction of the analyte with the bioreceptor into an electronic one. The intensity of signal is proportional to analyte concentration” [17]. Various bio-molecules (such as enzyme, antibody, oligonucleotide sequence, cells, etc.) have been used as bioreceptor. If biomolecular receptor is constituted by an aptamer, the biosensor is usually named as “aptasensor”.

The first step of the aptasensor development is represented by the immobilization of the aptamer capture probe onto a substrate. A great variety of methods for aptamer immobilization has been reported in literature. The probe immobilization step plays the major role in determining the overall performance of an aptasensor. The achievement of high sensitivity and selectivity requires maximization of the binding efficiency and minimization of non-specific adsorption, respectively. Control of the immobilization process is essential for assuring high reactivity, orientation, accessibility and stability of the surface-confined aptamer probe as well as for minimizing non-specific adsorption events. The choice of the immobilization method is dependent on the assay format and detection principle and strictly influence the ability of the aptamer to bind the target protein. The most successful approaches are the covalent bond, the affinity reaction and the self-assembled layer. In particular to guarantee suitable stability, surface coverage and maintaining the same binding affinity as showed in solution, various molecules (such as tetra(ethylene glycol), TEG, or –(CH_2_)_6_–, etc.) acting as arm spacers were introduced. 

After a proper blocking step, in order to avoid the non-specific adsorption of the interfering substances, affinity reaction with the target is performed. The assay format and aptasensing strategy are largely determined by the size of the target ligand.

**Figure 1:**
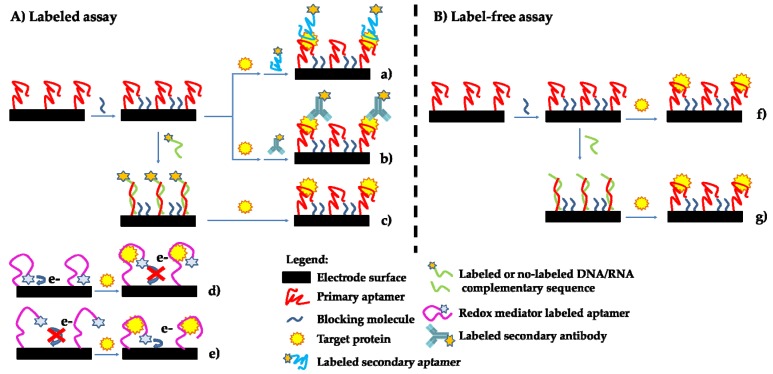
Schematic representation of aptamer-based sensor approaches. (**A**) Labeled assay: (**a**) sandwich, (**b**) mixed sandwich, (**c**) displacement, (**d**) switch-off, (**e**) switch-on; (**B**) label-free assay: (**f**) label-free, and (**g**) displacement.

Once the analyte has been captured onto the sensor surface, different strategies can be used for transducing the biorecognition event. The aptasensor can be divided into label and label-free format, as reported in Figure 1.

Labeling methods offer high sensitivity and specificity (Figure 1A). In sandwich format, the target molecule is captured between two aptamers (Figure 1Aa), or between an aptamer and an antibody (Figure 1Ab) that binds different regions of the target. The capture aptamer is anchored to the sensor surface, while the other labeled biorecognition element is used for detection. Labels include various kinds of molecules, such as enzymes (i.e., biotin/streptavidin-alkaline phosphatase conjugate) or electroactive compounds (i.e., ferrocene, gold nanoparticles), which can be directly bound to the secondary aptamer sequences during their synthesis. The sandwich approach allows the decrease of the non-specific signal in complex samples but the cost and the time of assay increase [18].

Since a DNA or RNA aptamer is able to bind its complementary sequence by hybridization reaction, aptamer-based displacement assays have also been reported (Figure 1Ac). In these assays, after the immobilization of the aptamer, a hybridization reaction with complementary labeled oligonucleotide sequence (i.e., enzyme, redox mediator, etc.) has been performed. The subsequent incubation with the analyte, which possesses higher affinity for the aptamer with respect to the complementary sequence, leads to the displacement of the complementary labeled target DNA/RNA sequence with the consequent decreasing of the analytical signal. 

Exploiting the 3D folding-based structure, two labeled strategies, named switch-on and switch-off, are described in literature. In both approaches, an aptamer functionalized with an electroactive molecule (i.e., ferrocene, methylene blue, etc.) is immobilized on the electrode surface. In the switch-on assay, after the binding reaction with the analyte (Figure 1Ad), the conformational change of the aptamer structure decreases the distance between the redox molecule leading to an increase of the measured electrochemical signal. In the case of switch-off strategy (Figure 1Ae), the distance between the electro-active molecule and the electrode surface increases and, thus, a decreasing of the electrochemical signal is observed. 

Among all the electrochemical techniques used for labeled assay, voltammetry (in which the current is measured in relation to a potential variation) plays a special role. In particular, because of the increasing of the signal-to-noise-ratio (due to the reduction of the background and capacitive current), which led to an increase of sensibility, voltammetric pulse techniques (such as differential pulsed voltammetry, DPV, and square wave voltammetry, SWV) have been widely applied.

The use of a label-based approach, coupled with pulsed voltammetric techniques, allows the decreasing of the non-specific signal (with the consequent increasing of the sensitivity) but with an increase of the cost and of the working time of the assay [19,20].

In the label-free approach, the aptamer-analyte binding modifies the interfacial properties of the aptasensor surface. Different electrochemical techniques, such as cyclic voltammetry, potentiometry, and electrochemical impedance spectroscopy, EIS (Figure 1Bf), can be used. In particular, in the EIS technique, after the application of a sinusoidal current or voltage to the electrode surface, a phase-shifted sinusoidal voltage or current was registered. By the variation of the frequency of the applied signal, the impedance (Z) of the electrode surface can be determined. Results were generally reported in the form of a Nyquist plot in which the real component of the impedance (Z’) is plotted against its imaginary part (−Z’’). An equivalent circuit (i.e., Randles circuit) is finally used to define the analytical information (mainly expressed in terms of double-layer capacitance, C_dl_, or of charge transfer resistance, R_ct_) regarding the bio-affinity reaction. In comparison with other techniques, EIS allows a proper sensitivity for the evaluation of bioreceptor-analyte interaction despite the use of a specific instrumentation and data analysis [21,22].

Label-free displacement assay using no-labeled complementary sequence has also been reported (Figure 1Bg). In this approach, the changing of the molecules bound to the aptasensor leads to an increase of the recorded capacitance or resistance values because of steric hindrance and electrostatic interactions. The advantage of the label-free strategy is the real-time evaluation of the analyte-bioreceptor binding [23,24,25].

Some specific examples of the aptamer-based assays can be found in [26,27].

### 2.2. Electrochemical Aptasensors

Electrochemical aptasensors are affinity biosensors, which use an immobilized aptamer on electrode surface for binding the analyte selectively. The binding is detected by change in currents and/or voltages at the localized surface. Based on their operating principle, the electrochemical biosensors can employ potentiometric, amperometric, impedimetric, and conductometric techniques converting the chemical information into a measurable signal [28,29,30]. 

In recent years, nanostructured materials have been extensively applied in electrochemical biosensors [31,32,33]. Label and label-free strategies have benefited in terms of sensitivity, biocompatibility, miniaturization, by peculiar characteristics of nanomaterials, such as high-energy surface and surface-to-volume ratio, surface catalytic effect, etc. Gold nanoparticles and rods (AuNPs and AuNRs, respectively), silver nanoparticles (AgNPs), magnetic nano-beads, multi-walled carbon nanotubes (MWCNTs), and nano-conductive polymers [34,35,36,37] are among the most-used nanomaterials. This paragraph focuses on the recent advances of aptasensors for cancer biomarker detection and monitoring, such as mucin 1, human epidermal growth factor receptor 2, prostate-specific antigen, carcinoembryonic antigen, vascular endothelial growth factor, and platelet-derived growth factors. An extended list of electrochemical aptasensors is reported in Table 1. Although, the most detected protein has been thrombin, it will not discussed in this section because several excellent reviews have been already published covering this issue [38,39,40].

Mucin 1 (MUC1) is a type I transmembrane glycoprotein (300–600 kDa) belonging to the mucins protein family; its soluble form, derived from proteolytic cleavage, is known as carbohydrate antigen 15.3. Both proteins can be mainly overexpressed in serum (CA 15-3 > 20 U/mL, MUC1 > 5 µM) in the presence of breast and ovarian carcinomas; elevated serum concentrations are always associated with a low patient survival rate [77]. MUC1 detection has been achieved by various aptasensor approaches, including sandwich, switch-on, switch-off, and label-free assays.

**Figure 2:**
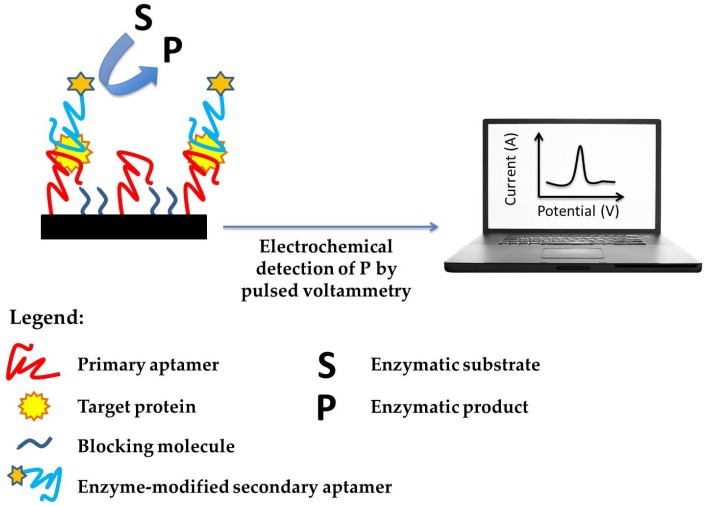
Schematic representation of aptamer-based electrochemical sandwich assay.

Florea et al. reported the development of a sandwich assay based on the use of magnetic beads (MBs) as a platform for aptamer immobilization [41]. In this work, streptavidin-modified MBs were modified with a biotinylated anti-MUC1 primary aptamer, followed by a blocking step with biotin and by the affinity reaction with the target protein. The sandwich assay was then completed by the incubation with a biotin-modified secondary anti-MUC1 aptamer and the use of streptavidin-alkaline phosphatase as an enzymatic label. The modified-beads were then collected on the surface of the working electrode with the help of a magnetic bar and incubated with alpha-naphthyl phosphate as the enzymatic substrate. Differential pulse voltammetry (DPV) measurements allowed the construction of a linear calibration curve in the range between 0.05 and 0.28 nM MUC1 with a detection limit of 0.07 nM. The authors also reported the high selectivity for MUC1 protein (using mucin 4 and mucin 16 as non-specific proteins) of the proposed aptasensor, with respect to the antibody sandwich-based assay and the ability to detect MUC1 in cancer patient serum samples (Figure 2). A generic schematic representation of an aptamer-based electrochemical sandwich assay was reported in Figure 2.

A switch-on-based aptasensor was reported by Hu et al. [44]. Biotin/thiol-modified anti MUC1 hairpin aptamer was firstly immobilized on the surface of ATP/horseradish peroxidase (HRP)/gold nanoparticles bioconjugate via a thiol-gold bond. The addition of MUC1 allowed the opening of the hairpin aptamer with the consequent exposure of the biotin to the surface of streptavidin-modified carbon glassy electrode. DPV detection of the 2,3-diaminophenazine (DAP) redox molecule (obtained from the oxidation of o-phenylenediamine, oPD, by H_2_O_2_ in the presence of HRP) allowed the detection of MUC1 protein in a linear range from 8.8 to 353.3 nM and a detection limit of 2.2 nM. The proposed switch-on aptasensor showed good selectivity for MUC1 protein detection in the presence of carcinoembryonic antigen (CEA) and myoglobin, and good recovery (ranging from 101.2% and 108.9%) in spiked serum samples.

Liu et al. proposed a gold nanoparticles-based assay [47]. Aptamer-modified AuNPs were incubated with a gold electrode functionalized with a DNA probe complementary to the aptamer sequence. After the addition of MUC1 protein, the displacement of the aptamer/AuNPs conjugation occurred and the increased charge transfer resistance values (R_ct_) by the electrochemical impedance spectroscopy technique were evaluated. This approach allowed the detection of MUC1 in a range between 0.5 and 10 nM, with a limit of detection of 0.1 nM. Selectivity in the presence of carcinoembryonic antigen, CEA, and tumor necrosis factor alpha, TNF-α, proteins was evaluated. Moreover, MUC1 fortified serum samples were also successfully analyzed.

Human epidermal growth factor receptor 2 (HER2), also known as ErbB2, c-erbB2, or HER2/neu, is a type 1 transmembrane glycoprotein (185 kDa) which belongs to a family composed of four structurally-related members, HER1 (ErbB1, also known as EGFR), HER2, HER3, and HER4. Overexpression of HER2 usually results in malignant transformation of cells, accounting for ~25% of all breast cancers (clinical serum cut-off 15 ng/mL) and it is always associated with more aggressive tumor phenotypes. HER2 can be also found at high concentration in blood in presence of gastric, ovarian, and prostate cancers [78].

Due to a couple of aptamers, which can bind two distinct parts of HER2 molecules has not been reported yet, HER2 detection is mainly performed by label-free or antibody/aptamer sandwich assay.

Qureshi et al. reported the development of a label-free aptasensor based on the use of a gold interdigitated electrode (IDE) [50]. The assay was based on the covalent immobilization of anti-HER2 DNA aptamer using 1-ethyl-3-(3′-dimethylaminopropyl)carbodiimide hydrochloride (EDAC) and *N*-hydroxysuccinimide (NHS) on the surface of a β-mercaptopropionic acid-modified IDE. The aptasensor was then incubated with HER2. The variation of the capacitance (at fixed frequency, 242 MHz) of IDE electrode allowed the detection of HER2 in spiked serum samples in a linear range between 0.2 and 2 ng/mL with a detection limit of 0.2 ng/mL. The proposed aptasensor also showed appropriated selectivity in the presence of epidermal growth factor receptor (EGFR) and vascular endothelial growth factor (VEGF).

Chun et al. used gold nanoparticle-modified electrodes as nanostructured surfaces [51]. The anti-HER2 aptamer was immobilized by EDAC/NHS amine coupling to the surface of a 3-mercaptopropionic acid (MPA)/AuNPs-modified gold electrode. After a blocking step, an affinity reaction with the protein was carried out and evaluated by means of electrochemical impedance spectroscopy (EIS) in terms of variation of R_ct_ (ΔR_ct_). Selectivity in the presence of various molecules, such as glucose, IgG, etc., was also evaluated.

Antibody/aptamer mixed sandwich-based assay was reported by Zhu et al. [52]. HER2 protein was sandwiched between a primary anti-HER2 antibody, immobilized on the surface of a self-assembly monolayer of 2,5-bis(2-thienyl)-1*H*-pyrrole-1-(p-benzoic acid) (DPB) supported on the AuNPs-modified glassy carbon electrode, and a secondary anti-HER2 aptamer was immobilized on the surface of hydrazine-modified AuNPs. After, the silver enhancement reaction was carried out by the addition of silver nitrate solution. In particular, Ag^+^ reduction to Ag° (in the presence of hydrazine as a reducing agent) was catalyzed by the AuNPs with the consequent formation of bi-metallic nanoparticles (Ag/AuNPs). The electrochemical detection of silver, by anodic stripping voltammetry, allowed the detection of HER2 in two linear ranges, between 0.0001 and 0.05 ng/mL and 0.1 and 10 ng/mL. The reported biosensor was also applied to in vivo applications, such as breast cancer cell determination (limit of detection 26 cells/mL) and imaging.

Prostate specific antigen (PSA) is one of the most used cancer biomarker, after the discovery in the mid-1980s of its potential role in screening and diagnosis of cancer prostate (CaP) (clinical cut-off 4 ng/mL). PSA is a 240 amino acid protein (34 kDa) belonging to the glandular kallikrein protein family. It is an approved cancer biomarker from the Food and Drug Administration; however, limitations have been found in cancer diagnosis. Only 25% of men with high PSA levels are affected by CaP because the PSA concentration could be elevated in the presence of benign conditions. To overcome this drawback, some modifications of PSA screening tests were introduced, i.e., the evaluation of PSA density or velocity (measuring the variation of PSA concentration vs. time, expressed as ng/mL/years), the correlation of the PSA level with age and between the amounts of free PSA (PSA_f_) and total PSA (PSA_t_) [79].

Kavosi et al. achieved the detection of PSA by the development of an antibody/aptamer mixed sandwich assay based on a triple amplification system [54]. At first, a graphene oxide/chitosan-modified glassy carbon electrode was functionalized with an anti-PSA antibody by means of glutaraldehyde and thionine. Then, the affinity reaction with PSA was carried out, followed by the incubation with a label (composed by AuNPs/polyamidoamine (PMAM) dendrimer particles functionalized with biotinylated and non-modified anti-PSA aptamers) and with streptavidin-modified horseradish peroxidase (HRP). Detection of PSA was achieved both by EIS, using [Fe(CN)_6_]^3−/4−^ as a redox mediator (linear range 0.05–35 ng/mL, detection limit 5 pg/mL) and, after the addition of H_2_O_2_, by DPV (linear range 0.1 pg/mL–90 ng/mL, detection limit 10 fg/mL). The proposed biosensor also showed good correlation with the ELISA reference method in the case of analysis of cancer tissues and human serum samples.

Souada et al. proposed a switch-on/off assay [56]. A quinone-based conductive polymer was electropolymerized on the surface of a glassy carbon working electrode followed by the immobilization of the anti-PSA aptamer. After the affinity reaction with PSA, a decrease on redox current, evaluated by means of SWV, was observed due to the steric hindrance generated on the surface of the electrode by the PSA/aptamer complex (switch-off). The following addition of a complementary DNA sequence caused the displacement of PSA protein leading to an increase of the observed current (switch-on). This approach allowed the detection of PSA in a linear range between 0.001 and 10 µg/mL, with a detection limit of 1 ng/mL.

A label-free aptasensor was proposed by Rahi et al. [57]. In this work, gold nanoparticles were electrodeposited on the surface of a gold electrode starting from a solution of HAuCl_4_ in presence of H_2_SO_4_ and arginine by means of cyclic voltammetry. The nanostructured electrode was then functionalized with a thiol-modified anti-PSA aptamer followed by the affinity reaction with the target protein and by the incubation with methylene blue as a DNA intercalated redox probe. In the presence of the protein, the displacement of methylene blue (MB) occurred with the consequent decrease of its redox current peak evaluated by SWV. This approach allowed the detection of PSA in a linear range between 0.125 and 200 ng/mL with a detection limit of 50 pg/mL in buffered solutions. The proposed aptasensor was then successfully applied for the detection of PSA in healthy and cancer patients’ serum samples.

Carcinoembryonic antigen (CEA) is a 180 kDa glycoprotein involved mainly in cell adhesion, and is one of the most used biomarkers for gastrointestinal cancer diagnosis, stage detection, and to evaluate the cancer recurrence after therapeutic treatments (clinical cut-off 2.5 µg/mL). CEA overexpression can be also related to the presence of lung, pancreatic, breast tumors, and to cancer metastatic processes [80,81].

A simple sandwich-based aptasensor for CEA detection was proposed by Shu et al. [60]. The CEA antigen was sandwiched between a primary aptamer (immobilized on the surface of a gold electrode) and a secondary aptamer immobilized on the surface of 6-ferrocenyl hexanethiol-modified AuNPs. CEA concentration in fortified human serum samples were evaluated by means of DPV measurements analyzing the behavior of a ferrocene redox probe (linear range 1–200 ng/mL, detection limit 0.5 ng/mL). The selectivity of the proposed biosensor in the presence of myoglobin, mucoprotein, and bovine serum albumin was also evaluated. 

An antibody/aptamer mixed assay was also reported [61]. In this work, graphene dispersion was functionalized with an anti-CEA antibody, dropped on the surface of glassy carbon electrode, and left to dry. The assay was then performed by incubation with CEA protein and silver nanoclusters (AgNCs) functionalized with anti-CEA aptamer and HRP. The enzyme, in the presence of H_2_O_2_, catalyzed the polymerization of 3,3′-dimethoxybenzidine creating an insulating layer on the electrode surface which reduced the redox current of the [Fe(CN)_6_]^3−/4−^ redox mediator. The CEA protein was detected by SWV in a linear range between 1 and 10,000 ng/mL with a detection limit of 0.5 pg/mL. The proposed aptasensor showed good results in analysis of serum samples in comparison with the ELISA method.

A label-free approach, based on a ternary nanocomposite made of AuNPs, hemin, and graphene, was reported by Liu et al. [63]. This nanocomposite material was assembled on the glassy carbon electrode surface and functionalized with the anti-CEA thiolated aptamer. Affinity reaction with CEA protein was then carried out and evaluated by means of DPV measurements, analyzing the decreasing of phosphate peak current, in a range between 100 and 10^6^ fg/mL with a detection limit of 40 fg/mL. The label-free aptasensor showed good selectivity in the presence of human serum albumin, thrombin, lysozyme, insulin, and good recovery values (ranging from 97% to 99.5%) in the case of CEA-spiked serum samples analysis.

Vascular endothelial growth factors (VEGF) are a family of dimer glycoprotein, which includes five members (VEGF-A, VEGF-B, VEGF-C, VEGF-D, and placenta growth factor, PGF) and their associated receptors (VEGFR-1, VEGFR-2, VEGFR-3). Since VEGF-A was the first and the most-studied cancer protein, often, the term VEGF strictly indicates the VEGF-A member (which can also be distinguished based on the number of constituent amino acids in VEGF_121_, VEGF_165_ and VEGF_189_) [82].

Platelet-derived growth factor (PDGF) is another 30 kDa dimer glycoprotein (composed by two A, PDGF-AA, or two B, PDGF-BB, chains or the combination of the two, PDGF-AB) member of the growth factor family protein [83]. Since both growth factors are involved in cancer angiogenesis and vascularization processes, their overexpression in serum (VEGF > 100 pM; PDGF > 40 ng/mL) can be related to the presence of cancer metastasis process [84].

In our previous work [64], we reported the development of an aptamer sandwich-based assay for the detection of VEGF-A cancer protein by the use of gold nanostructured graphite screen-printed electrodes. Gold nanoparticles were firstly electrodeposited onto the electrode surface from a HAuCl_4_ acid solution and then functionalized with the thiolated primary anti-VEGF aptamer. After the formation of a mixed SAM (using 6-mercapto-1-hexanol) and a proper surface blocking step, the affinity reaction with VEGF was carried out followed by the incubation with a biotinylated secondary anti-VEGF aptamer. After the addition of streptavidin-alkaline phosphatase and of alpha-naphthyl phosphate as the enzymatic substrate, the oxidation of the enzymatic product (alpha-naphthol) was evaluated by DPV measurements. The developed assay was able to detect VEGF protein in a linear range between 40 and 250 nM with a detection limit of 30 nM.

Switch-off and label-free strategies were reported by Shamsipur et al. [67]. In both strategies, a glassy carbon electrode was functionalized with gold nanoclusters and then modified with glutaraldehyde and with an anti-VEGF aptamer. In the case of the switch-off approach, the sensor surface was incubated with methylene blue (MB) as the redox probe, followed by the incubation with the target molecule. The addition of VEGF caused the displacement of MB with the subsequent decrease of the electrochemical signal (linear range 1–120 pM, detection limit 0.32 pM). In the label-free approach, after the functionalization with the anti-VEGF aptamer, the sensor was directly incubated with the protein. The variation of charge transfer resistance values (ΔR_ct_), measured by EIS, allowed the detection of the cancer protein in a linear range of 2.5–500 pM with a detection limit of 0.48 pM. Analysis of a serum sample from a healthy patient, and comparison with an ELISA test, were finally performed to prove the applicability of the proposed aptasensor.

Various approaches were also used for the detection of the PDGF-BB cancer protein, using different nanomaterials. Fang et al. [68] reported a sandwich assay based on AuNPs and a molybdenum disulfide/carbon aerogel-modified electrode (linear range 0.001–10 nM, detection limit 0.3 pM).

Song et al. exploited the in situ aggregation of AgNPs functionalized with the anti-PDGF aptamer induced by the hybridization of an immobilized DNA probe. The subsequent detection of Ag by DPV allowed the estimation of the protein in the range between 5 and 1000 ng/mL with a detection limit of 1.6 pg/mL [70]. 

Label-free electrochemical aptasensors for PDGF detection were also reported [74,75,76]. He et al. proposed the use of Co_3_(PO_4_)_2_-anti-PDGF aptamer nanocomposite, which was immobilized on the surface of a gold electrode. After the affinity reaction with PDGF, EIS measurements allowed the detection of the target protein in a linear range between 0.01 and 100 ng/mL with a detection limit of 3.7 pg/mL [74]. Gold nanoparticles/leaf-like VS_2_ nanosheet-modified glassy carbon electrodes were used instead by Liu et al. as a sensing platform [75]. In this work, the anti-PDGF aptamer was immobilized on the surface of the nanostructured electrode and incubated with the protein. The evaluation of [Fe(CN)_6_]^3−/4−^ current decreasing by DPV due to the increasing of PDGF concentration onto the electrode surface (linear range 0.001–1 nM, detection limit of 0.4 pM). The proposed aptasensor also showed good selectivity (in the presence of IgE, hemoglobin, thrombin, and BSA) and good comparison with ELISA tests, in the case of urine sample analysis. Finally, Deng et al. [76] reported the employment of poly(diallyldimethylammonium chloride) (PDDA)-protected graphene-gold nanoparticles (P-Gra-GNPs) modified with anti-PDGF aptamer, using glucose oxidase (GOD) both as a surface blocking agent and as a sensing probe. In fact, in the presence of PDGF protein, the decrease of the enzyme direct electron transfer (DET) on FAD/FADH_2_ redox reaction (due to the increasing of spatial blocking around the GOD) was evaluated by means of CV measurements (linear range 0.005–60 nM; detection limit 1.7 pM). Selectivity and stability of the proposed label-free aptasensor were also studied.

### 2.3. Electrochemiluminescence Aptasensors

Electrogenerated chemiluminescence or electrochemiluminescence (ECL) is an example of luminescence. Luminescent transitions occur during relaxation from an excited intermediate level to a lower energy level state. These transitions are characterized by dissipation of radiations as photons in the UV, visible, and close IR region. Light can be emitted during a reaction in a number of different ways: from chemical reactions as chemiluminescence, from an external radiation as photoluminescence, from electrochemical reactions as electrogenerated luminescence and from biological systems as bioluminescence. ECL detection consists in monitoring the production of photons and, thus, the light intensity produced during the electrochemical reaction in solution. Therefore, light intensity is directly proportional to the concentration of one or all the reactants involved in the electrochemical reaction [85].

**Figure 3:**
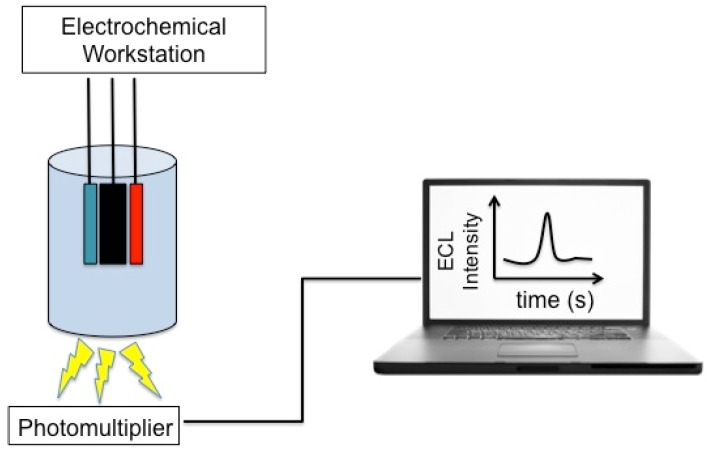
Schematic representation of the general instrumentations of electrochemiluminescence bioanalysis. Adapted with permission from [112]. ©2015, American Chemical Society.

Schematic representation of the general instrumentations of electrochemiluminescence bioanalysis is reported in Figure 3. The species generated at electrodes undergo high-energy electron-transfer reactions to form excited states that emit light, which is then acquired by a photomultiplier.

One of the most reported analytes in the ECL-based aptasensor literature, in the last decade, is thrombin. The first aptamer binding to human α-thrombin was described by Bock et al. in 1992 [86]. This 15-mer DNA oligonucleotide (5′-GGT TGG TGT GGT TGG-3′) can form a stable intramolecular G-quadruplex structure, with a dissociation constant (K_d_) of 100 nM [87]. Another thrombin binding aptamer is a 29-mer DNA oligonucleotide (5′-AGT CCG TGG TAG GGC AGG TTG GGG TGA CT-3′), which binds to the heparin-binding exosite of thrombin with a higher affinity (K_d_ = 0.5 nM) in a G-quadruplex structure [88]. The two aptamers can bind on two distinct binding sites of thrombin without interfering with each other’s binding, giving them high advantages in biosensors development. Some examples of aptasensors for thrombin detection have been reported in literature, and many of them are listed in Table 2.

As a general comment, to explain the number of papers dealing on thrombin detection, with respect to other biomarkers, it should be noted that thrombin aptamers are robust and well characterized bioreceptors [12]. For this reason, they have been used as models to develop innovative ECL-detection schemes, involving new electrode materials and innovative nanomaterial-based emitters. Recently, an aptasensor based on surface-initiated-atom-transfer-radical-polymerization (SI-ATRP), to facilitate high-density immobilization of luminophores, and manganese dioxide-graphene (MnO_2_-GO) composite (to indirectly deactivate the excited state of [Ru(bpy)_3_]^2+^ for ultrasensitive detection of carcinoembryonic antigen (CEA), was proposed [89]. In this approach, MnO_2_-GO composite served as an efficient quencher for indirect deactivation of the excited state of [Ru(bpy)_3_]^2+^. SI-ATRP was applied to functionalize multiwalled carbon nanotubes (MWNTs) with glycidyl methacrylate (GMA) as the functional monomer. A nanocomposite material of polyamidoamine (PAMAM) dendrimer encapsulated AuNPs was used as the carrier to combine [Ru(bpy)_3_]^2+^ and poly-GMA together for the synthesis of the ECL matrices. The prepared matrices were applied to bind amino-modified auxiliary probe I (A1), which was partially complementary with the CEA aptamer. Meanwhile, the MnO_2_–GO composite was modified with another amino-modified CEA aptamer-partial-complementary auxiliary probe II (A2). Through the hybridization of the CEA aptamer with A1 and A2, the quencher MnO_2_-GO composite was linked with the ECL matrices, by which a low ECL signal was detected (off-state). However, in the presence of CEA, the sandwich-like structure was destroyed because CEA would bind to its aptamer in lieu of the auxiliary probes, which resulted in a recovery of the ECL signal (on-state). The proposed ECL aptasensor showed high sensitivity with a detection limit of 25.3 fg/mL and a wide linear range of 0.1–20 ng/mL.

Examples of electrochemiluminescence aptasensor for platelet-derived growth factor B chain (PDGF-BB) and MUC1 can be found in [90,91], respectively. In both case *N*-(aminobutyl)-*N*-ethylisoluminol (ABEI) was used as emitter.

### 2.4. Photoelectrochemical Aptasensors

The process of photoelectrochemical (PEC) detection can be discussed as the reverse of the ECL process [113,114,115,116]. Indeed, PEC detection consists in monitoring the production of a photocurrent, using the light as the excitation source [117]. Photoelectrochemistry is based on the use of photoactive material, generally inorganic, organic, or hybrid semiconductors. Inorganic semiconductors can be made conductive either by putting extra electrons into the conduction band (CB) or by removing electrons from the valence band (VB). Generally, it is due to the movement of electrons through the mostly empty CB. Removal of an electron from the VB creates a positively-charged vacancy called a hole. Electrons can be promoted from the VB to the CB upon the absorption of photons of light and, thus, a necessary condition is that photon energy exceeds the band gap energy (hν > E_band gap_). An anodic photocurrent occurs when the CB electrons are transferred to the electrode, and VB holes neutralized by electrons supplied by an electron donor D in solution [118]. If the CB electrons are transferred to a solution-solubilized electron acceptor, a cathodic photocurrent [116] is measured. The presence of an efficient electron donor/acceptor prevents the electron-hole recombination and, thus, increases and stabilizes the photocurrent.

Nowadays, inorganic semiconductors are mainly fabricated using manifold nanomaterials like SnO_2_, TiO_2_ nanoparticles (NPs), as well as CdS and CdSe quantum dots (QDs). Frequently, the photoanode is assembled by depositing nanomaterials on a conductive substrate, such as gold, indium-tin-oxide (ITO), or F-doped SnO_2_ (FTO). On the other hand, organic semiconductors include small molecules such as porphyrin, phtalocyanine, and their derivatives, azo dyes, metal complexes, as well as polymers. The light excites the molecules that can react with an electron donor or an electron acceptor, producing anodic or cathodic photocurrents, respectively. In addition, in this case, organic semiconductors are deposited on a conductive substrate.

Hybrid semiconductors can be obtained by coupling two inorganic semiconductors with different band gaps or organic complexes combined with inorganic materials; in this sense improved conversion efficiency is obtained by coupling semiconductors with different band gaps to control the charge separation and retard electron/hole recombination [117]. Furthermore, various bulk electrodes, such as gold or ITO electrodes, as well as TiO_2_ nanotubes, could be employed directly in the assessment of the photocurrent.

Recently, AuNPs or carbon nanostructures are highly used in combination with semiconductors. Some examples are AuNPs/TiO_2_ hybrid materials, graphene/CdSe, and porphyrin/fullerene/AuNPs. In conclusion, any materials with good photoresponsability might be used in PEC detection [117].

**Figure 4:**
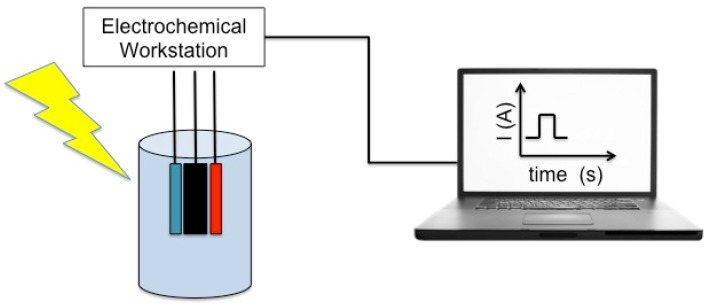
Schematic representation of the general instrumentations of photoelectrochemical bioianalysis. Adapted with permission from [112]. © 2015, American Chemical Society.

PEC detection offers some peculiar advantages of the electrochemical detection, i.e., low cost, simple and easy to miniaturize instrumentation, etc. Furthermore, by using light for excitation and a separate form of detection, PEC possesses potentially high sensitivity because of the reduced background associated with it. A schematic representation of general photoelectrochemical apparatus for bioanalysis is reported in Figure 4.

According to the mode of signal transduction, PEC biosensors could be organized in two modes: potentiometric and amperometric [117]. In this review, we will focus only on amperometric detection.

Label and label-free formats are reported and characterized by different processes in the signal generation.

In the case of label-based formats, the biorecognition event is accomplished with the presence of a label (Au NPs, QDs, graphene, enzymes, etc.) that modulates the photocurrent formation. In the label-free format, the biorecognition event would break the original balance and influence the photocurrent. For instance, the analyte is oxidized by the photogenerated holes and the recombination of photogenerated electrons and holes is inhibited [119]; moreover, the biorecognition event hinders the diffusion of the sacrificial electron donor, inducing a photocurrent decrease [120].

Some examples of aptasensors for clinical biomarker detection, by using PEC detection, are reported in Table 3, together with their limit of detection. Among other biomarkers, different strategies for CEA detection have been reported. Zeng et al. [121] described a method based on resonance energy transfer (RET). This phenomenon has been applied to biosensing with various detection techniques, including fluorescence, surface plasmon resonance (SPR), and electrochemiluminescence (ECL). Zeng et al. [121] developed a PEC platform based on RET between CdTe QDs and RGO-AuNPs nanocomposites.

To this end, an ITO electrode was modified using mercaptoacetic acid-wrapped CdTe (MPA-CdTe) QDs; under visible light (470 nm) excitation, a cathodic photocurrent was obtained at negative bias potential (−0.05 V). Resonance energy transfer caused by excition-plasmon resonance between reduced graphene oxide (RGO)-Au nanoparticles (AuNPs) and CdTe QDs was obtained. Upon the sandwich-like structure formed via DNA hybridization, the exciton produced in CdTe QDs was annihilated. A damped photocurrent was obtained, which acted as the background signal for the development of the PEC platform. When CEA bound to its specific aptamer and destroyed the sandwich-like structure, the energy transfer efficiency was lowered, leading to an increase in the photocurrent. A limit of detection of 0.47 pg/mL was reported.

In Table 3 some examples of circulating cell detection are also reported. Recently, circulating tumor cells (CTCs) have received enormous attention as new biomarkers [122] and different detection schemes have been proposed for their detection, including photoelectrochemistry.

## 3. Conclusions

In the past decade, the interest in the development of aptamers against biomarkers has increased remarkably. Aptamers have demonstrated enough merits and potentials in biosensor field. The use of nanomaterials in the development of aptamer-based biosensors for biomarker diagnosis improves the development of highly-versatile diagnostic devices that are small, portable, easy to use, low in cost, and disposable. This will make aptamer-based biosensors more and more interesting for application in clinical analysis.

## Figures and Tables

**Table 1:** Analytical characteristics of electrochemical aptasensors for cancer biomarkers detection.

Biomarker	Assay Strategy	Signal Amplification	Electrochemical Technique	LOD	Reference
MUC1	Sandwich	Enzymatic	DPV	0.07 nM	[41]
	Sandwich	AuNPs/MWCNTs	DPV	1 pM	[42]
	Mixed sandwich	MB	DPV	0.62 ng/mL	[43]
	Switch-on	AuNPs	DPV	2.2 nM	[44]
	Switch-off	−	CV	50 nM	[45]
	Displacement	Exonuclease	SWV	4 pM	[46]
	Displacement	AuNPs	EIS	0.1 mM	[47]
	*Cells*				
	Sandwich	AuNPs	Chronoamperometry	8 cells/mL	[48]
	Label-free	CNSs	EIS	40 cells/mL	[49]
HER2	Label-free	−	EIS	0.2 ng/mL	[50]
	Label-free	AuNPs	EIS	10^−5^ ng/mL	[51]
	Mixed sandwich	AgNPs/AuNPs	SWV	0.037 pg/mL	[52]
				26 cells/mL	
PSA	Mixed sandwich	AuNPs	DPV	0.02 fg/mL	[53]
	Mixed sandwich	AuNPs/PAMAM dendrimer	DPV	10 fg/mL	[54]
			EIS	5 pg/mL	
	Displacement	CNTs/Chitosan	DPV	0.74 ng/mL	[55]
	Switch on/off	−	SWV	1 ng/mL	[56]
	Label-free	Au nanospears	DPV	50 pg/mL	[57]
	Label free	−	EIS	0.5 pg/mL	[58]
	Label free	−	EIS	< 1 ng/mL	[59]
CEA	Sandwich	AuNPs	DPV	0.5 ng/mL	[60]
	Mixed sandwich	AgNCs	SWV	0.5 pg/mL	[61]
	Label-free	PPY/CNTs	FET	1 fg/mL	[62]
	Label free	AuNPs/HGNs	DPV	40 fg/mL	[63]
VEGF	Sandwich	AuNPs	DPV	30 nM	[64]
	Mixed sandwich	−	Capacitance	From 400 to 800 pg/mL	[65]
	Label-free	mesoporous carbon/gold nanocomposite	EIS	1 pg/mL	[66]
	Switch-off	AuNCs	DPV	0.32 pM	[67]
	Label-free		EIS	0.48 pM	
PDGF-BB	Sandwich	AuNPs	DPV	0.3 pM	[68]
	Sandwich	PAMAM dendrimer	DPV	0.02 pM	[69]
	Sandwich	AuNPs/AgNPs	DPV	1.6 pg/mL	[70]
	Switch-on	Endonuclease	CV	10 pg/mL	[71]
	Displacement	Endonuclease	DPV	20 fM	[72]
	Displacement	−	DPV	1.6 fM	[73]
	Label-free	Co_3_(PO_4_)_2_ nanocomposites	EIS	3.7 pg/mL	[74]
	Label-free	AuNP and VS_2_ nanosheet	EIS	0.4 pM	[75]
	Label-free	Graphene/AuNPs	CV	1.7 pM	[76]

MUC1: mucin 1; HER2: human epidermal growth factor receptor 2; PSA: prostate-specific antigen; CEA: carcinoembryonic antigen; VEGF: vascular endothelial growth factor; PDGF: platelet-derived growth factors; AuNPs: gold nanoparticles; AuNCs: gold nanoclusters MWCNTs: multi-walled carbon nanotubes; MB: methylene blue; CNSs: carbon nanospheres; AgNPs: silver nanoparticles; PAMAM: polyamidoamine; CNTs: carbon nanotubes; MIP: molecular imprinted polymer; AgNCs: silver nanoclusters; PPY: polypyrrole; HGNs: hemin graphene nanosheets. DPV: differential pulse voltammetry; CV: cyclic voltammetry; SWV: square wave voltammetry; EIS: electrochemical impedance spectroscopy; FET: field effect transistor.

**Table 2:** Analytical performances of ECL-based aptasensors for the detection of thrombin.

Luminophore	LOD	Reference
CdTe	0.03 fM	[92]
Ru complex and pNAMA-HGNPs hydrogel composites	0.54 fM	[93]
Europium and MWCNT	0.23 pM	[94]
CdSe	2.7 aM	[95]
AuNPs/TSC-PTC/C60	3.3 fM	[96]
AuNPs-CdSeTe-ZnS	0.28 fM	[97]
Eu^3+^-doped CdS nanocrystals	1 aM	[98]
Ruthenium(II) complex	2.0 × 10^−15^ M	[99]
Ru(bpy)_3_^2+^/Dpa-mel CNSs	2.2 × 10^−13^ M	[100]
Ru(phen)_3_^2+^	1.2 aM	[101]
PAMAM / Ru(II) complex	5.0 fM	[102]
GDH and hemin/G-quadruplex	33 fM	[103]
tris(bipyridine) Ru(II)-β cyclodextrin	0.1 pM	[104]
HGNPs/GOxNPs/PtNPs	0.3 fM	[105]
Ru(phen)_3_^2+^	0.4 pM	[106]
Ir(III) complex	1.3 nM	[107]
luminol-AuNPs	1.7 pM	[108]
CdS:MnNCs and CdTe/SiO_2_ NPs	1 aM	[109]
CdS thin films and AuNPs	100 aM	[110]
Ruthenium complex	3 × 10^−15^ M	[111]

AuNPs: Au nanoparticles; Dpa-mel CNS: dopamine-melanin colloidal nanospheres; HGNPs: hollow gold nanoparticles; GDH: glucose dehydrogenase; GOxNPs: glucose oxidase nanoparticles; MWCNT: Multiwalled Carbon Nanotubes; NCs: nanocrystals; pNAMA: poly(*N*-(3-aminopropyl)methacrylamide); PtNPs: Pt nanoparticles; TSC-PTC: 3,4,9,10-perylene tetracarboxylic acid thiosemicarbazide.

**Table 3:** Analytical characteristics of photoelectrochemical aptasensors for biomarker detection.

Biomarker	Label and Signal Generation Process	LOD	Photoactive Materials	Reference
CEA	RGO-AuNPs nanocomposites in RET process	0.47 pg/mL	CdTe/ITO	[121]
	HRP impeding the light absorbance and B-4-CHD inhibiting AA diffusion to the electrode surface	1.38 pg/mL	CdSe/TiO2/RGO/ITO	[123]
MUC1	CdTe	0.52 nM	TiO_2_ nanotube arrays	[124]
thrombin	Ru(NH_3_)_6_^3+^	1 pM	Graphene-CdS/PEI/ITO	[125]
		-	CdSe/PAA-Graphene/PDDA/ITO	[126]
	Label-free; the analyte hinders the diffusion of the AA, inducing a photocurrent decrease	1 × 10^−13^ mol/L	(NTA-pyrene) and (Ru(II)-pyrene) complex	[120]
	AuNPs–glucose oxidase	1.9 × 10^−13^ mol/L	TiO_2_	[127]
	Label-free	1.2 × 10^−13^ mol/L	g-C_3_N_4_/TiO2/ITO	[128]
SMMC-7721 human hepatoma carcinoma cells	Label-free; steric hindrances for the diffusion of AA to the surface of CdSe	5.0 × 10 ^3^ cells/mL	CdS-PAMAM nano-composite/ITO	[129]
Ramos cell	Label-free; steric hindrances for the diffusion of AA to the surface of CdSe	84 cells/mL	CdSe/PDDA/ITO	[130]

FTO: F-doped SnO_2_; GO: graphene oxide; g-C_3_N_4_: graphitic carbon nitride; ITO: indium tin oxide; NTA: pyrenebutyric acid *N*α′,*N*α-bis(carboxymethyl)-l-lysine amide; PAMAM: polyamidoamine; PDDA: poly(dimethyldiallylammonium chloride); RET: resonance energy transfer; TEPA: tetraethylene pentamine; RGO: reduced graphene oxide.
